# Adjustable bandgap of a type-II AsP/SnS_2_ van der Waals heterostructure using strain: outstanding electronic, optical, and photocatalytic properties

**DOI:** 10.1039/d5ra07850d

**Published:** 2025-12-10

**Authors:** Dahua Ren, Yao Wen, Zhangyang Zhou, Ming Du, Liushun Wang, Jinqiao Yi

**Affiliations:** a College of Intelligent Systems Science and Engineering, Hubei Minzu University Enshi 44500 China nanx1013@163.com; b School of Physics and Technology, Wuhan University Wuhan 430072 China

## Abstract

In recent years, vertically stacked van der Waals heterostructures have attracted significant attention due to their highly tunable electronic properties and exceptional optical performance. Within the framework of density functional theory, we conducted a systematic investigation of the structural, electronic, and optical properties of a vertically stacked AsP/SnS_2_ heterostructure. Our calculations reveal that the AsP/SnS_2_ heterobilayer possesses an indirect bandgap of 1.24 eV, as determined by HSE06, and features a type-II band alignment that promotes the spatial separation of photogenerated charge carriers. Furthermore, the electronic structure of the AsP/SnS_2_ heterostructure is found to be highly sensitive to external strain, offering avenues for band engineering. Notably, the heterostructure exhibits markedly enhanced optical absorption in the visible range compared to its constituent monolayers. These results provide the potential of the AsP/SnS_2_ heterostructure as a promising candidate for high-performance visible-light-responsive photocatalysis and photoelectronic devices.

## Abstract 2

In recent years, vertically stacked van der Waals heterostructures have attracted significant attention due to their highly tunable electronic properties and exceptional optical performance.

## Graphical abstract

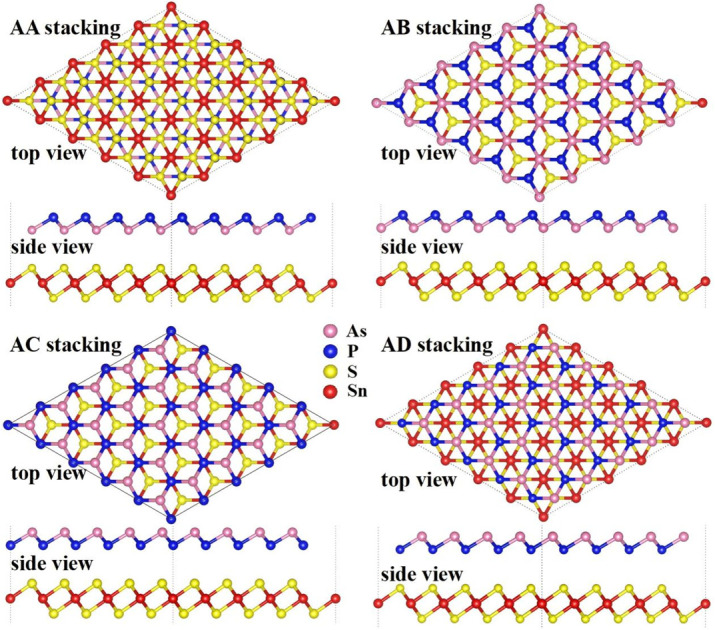

## 1 Introduction

van der Waals (vdW) heterostructures have emerged as a versatile platform for designing novel electronic devices by leveraging their unique physical properties to achieve exceptional performance.^1^ Vertically stacked heterostructures, in particular, have enabled innovative applications in electronics, including ultrathin photodetectors,^2^ solar cells,^3^ memory devices,^4^ flexible sensors and transistors.^5,6^ Among these, two-dimensional (2D) vdW heterostructures with a type-II band alignment show particular promise for use in photovoltaic and photocatalytic systems^7–11^ owing to their compelling electronic and optical properties. In such type-II heterostructures, photogenerated holes and electrons are spatially separated into distinct layers. This segregation effectively suppresses carrier recombination, thereby greatly improving the efficiency of light energy utilization.^12^

Moreover, two-dimensional arsenic-phosphorus (AsP) has recently emerged as a novel class of 2D materials; monolayer β-AsP is a typical honeycomb network structure that offers a suitable band gap (2.52 eV) and high carrier mobility.^13^ Both theoretical and experimental studies, including density functional theory (DFT) calculations, have demonstrated that the AsP monolayer exhibits a bandgap modulation from 0.20 eV to 2.20 eV, covering spectral ranges from visible to infrared regions and electron mobilities of up to 10^4^ cm^2^ V^−1^ s^−1^*via* strain engineering, external electric field application, or heterostructure formation.^13–18^ Monolayer AsP has the ability to form heterostructures with other 2D materials to enhance charge separation and functional performance; for example, the type-II β-AsP/SiC heterostructure is a semiconductor with a band gap of 1.65 eV.^13^ Strain engineering modifies the crystal field and orbital overlap; it involves applying mechanical stress (tensile or compressive) to a material to deform the crystal lattice, thereby altering the bond lengths and angles between atoms. Changing the distance between atoms affects the energy levels of the valence and conduction bands. Compressive strain pushes atoms closer together to typically increase the overlap between atomic orbitals, widening the bandgap and increasing the degeneracy of energy bands. Tensile strain pulls atoms apart to decrease orbital overlap, which generally narrows the bandgap. Zhang *et al.* demonstrated that monolayer honeycomb AsP becomes a direct band gap semiconductor with a band gap of 1.28 eV under 10% tensile strain along the biaxial direction, and the application of an electronic field also reduced the band gap.^14^ Biaxial strain refers to strain applied uniformly in two in-plane directions, *e.g.*, growing a thin film on a substrate with a different lattice constant. Uniaxial strain denotes strain applied along a single crystal direction, *e.g.*, bending a flexible substrate. For example, a slight compression (−2%) of the β-AsP/g-C_6_N_6_ heterostructure was found to be conducive to the hydrogen evolution reaction compared to monolayers.^17^ This tunability renders AsP highly promising for applications in photonics, electronics, and sensing, with demonstrated potential in mid-infrared photodetectors, thermoelectric generators, and lithium-ion batteries.

On the other hand, tin disulfide (SnS_2_) has garnered significant interest in solar energy conversion, optoelectronics, and photocatalysis due to its CdI_2_-type layered structure, moderate band gap (2.2–2.35 eV), and high electrical conductivity.^19^ Each layer of SnS_2_ consists of an S–Sn–S trilayer held together by van der Waals forces. Monolayer SnS_2_, typically produced *via* liquid exfoliation, exhibits a band gap of approximately 2.29 eV and shows promising performance in visible-light-driven water splitting owing to its high photoconversion efficiency.^20^ However, the inherent band edge alignment of pristine SnS_2_ limits its efficacy as a standalone photocatalyst for water splitting.^21^ Recent studies have shown that band engineering can be effectively achieved through various strategies, such as doping,^22^ external electric fields,^23^ and heterostructure formation.^24^

To date, several SnS_2_-based heterostructures have been explored to enhance their photocatalytic performance, including composites with MoS_2_,^24^ g-C_3_N_4_,^25^ SnO_2_,^26^ reduced graphene oxide (RGO),^27^ and ZrS_2_.^28^ Additionally, heterostructures incorporating SnS_2_, such as SnS_2_/BiPO_4_,^29^ have been engineered to achieve a type-II band alignment, yielding improved electronic and optical behavior for photocatalytic and optoelectronic applications. Despite these advances, the specific role and evolution of SnS_2_ within an AsP/SnS_2_ heterostructure remain unexplored. The integration of AsP and SnS_2_ into a van der Waals heterostructure represents a cutting-edge strategy that combines their complementary characteristics to achieve superior electronic and optoelectronic performance. Similar to analogous systems, such as the As/SnS_2_ heterostructure, which exhibits a type-II band alignment, efficient charge transfer, and strong absorption from visible to ultraviolet wavelengths, the AsP/SnS_2_ system is anticipated to facilitate efficient electron–hole separation and enhanced light harvesting. The bandgap tunability of AsP, coupled with the strong optical response of SnS_2_, can enable tailored heterostructures for broad-spectrum photodetection and high-efficiency photocatalysis. Strain engineering further offers a means to modulate electronic properties. However, the realization of high-quality, large-area AsP/SnS_2_ heterostructures requires overcoming substantial challenges, including lattice mismatch, interfacial defect control, and the development of scalable synthesis methods beyond mechanical exfoliation, such as chemical vapor deposition (CVD) and molecular beam epitaxy (MBE). Future research should focus on optimizing synthesis parameters, probing interfacial charge dynamics through advanced characterization and DFT simulations, and elucidating the influence of defects and doping on device performance. Success in these areas will establish AsP/SnS_2_ heterostructures as a transformative platform for next-generation devices, including low-power electronics, high-sensitivity photodetectors, and efficient energy conversion systems.

In this work, we constructed a novel 2D AsP/SnS_2_ van der Waals heterostructure and systematically investigated its structural, electronic, optical, and photocatalytic properties. The formation of a stable heterostructure is facilitated by the comparable lattice constants and structural compatibility between monolayer SnS_2_ and AsP. We further examined the exceptional structural and electronic characteristics of the AsP/SnS_2_ heterostructure, underscoring its potential as a high-performance material for photocatalysis and optoelectronic applications.

## 2 Computational methods

*Ab initio* calculations were carried out within the framework of density functional theory (DFT)^30,31^ using the Vienna *Ab Initio* Simulation Package (VASP).^32^ The exchange-correlation effects were treated using the Perdew–Burke–Ernzerhof (PBE)^33^ functional under the generalized gradient approximation (GGA),^34^ as well as with the HSE06 (ref. 35) hybrid functional, for improved electronic structure description. The electron-ion interactions were modeled using the projector-augmented wave (PAW) method.^36,37^ A plane-wave energy cutoff of 500 eV was employed, and the Brillouin zone was sampled with an 11 × 11 × 1 *k*-mesh for structural relaxation. For density of states (DOS) and electronic band structure calculations, a finer *k*-mesh of 13 × 13 × 1 was adopted. The energy and force convergence criteria were set to 10^−5^ eV and 10^−3^ eV Å^−1^, respectively. A vacuum layer thickness of at least 25 Å was included along the non-periodic direction to prevent spurious interactions between periodic images.^38^ Given the significance of long-range van der Waals (vdW) interactions in stabilizing the heterostructure, the DFT-D3 correction method^39^ was employed to account for these effects.

The optical properties of the AsP/SnS_2_ heterostructure are characterized by the complex dielectric function *ε*(*ω*) = *ε*_1_(*ω*) + *iε*_2_(*ω*), where *ε*_1_ and *ε*_2_ denote the real and imaginary parts, respectively. The imaginary component *ε*_2_(*ω*) is computed by summing all possible electronic transitions between the occupied and unoccupied states, reflecting the joint density of states and momentum matrix elements.^40–42^ It is given by:1
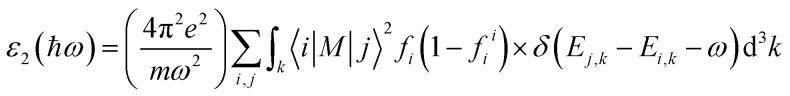
where *e* and *m* are the electron charge and mass, respectively; *ω* is the frequency of the incident photon; *M* is the dipole operator; *i* and *j* represent the initial and final state wavefunctions, with corresponding Fermi–Dirac occupation factors *f*_*i*_ and *f*_*j*_; and the integral is over the Brillouin zone. The real part *ε*_1_(*ω*) is derived from *ε*_2_(*ω*) using the Kramers–Kronig relationship:^41,42^2
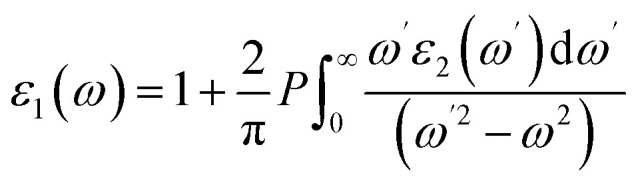
where *P* denotes the Cauchy principal value. The optical absorption coefficient *α* is subsequently obtained as3
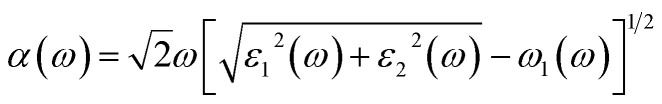


## 3 Results and discussion

### 3.1 Stability and structures

**Fig. 1:**
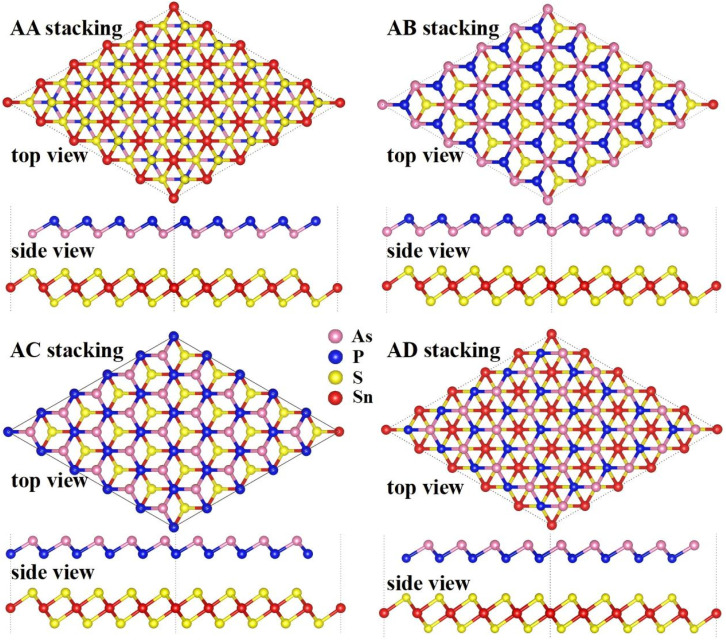
Relaxed structures (5 × 5 supercell) of the four typical stacking patterns of the AsP/SnS_2_ heterostructure.

The electronic properties of van der Waals heterostructures are highly sensitive to their stacking configuration. To systematically evaluate this effect, four distinct stacking patterns, denoted as AA, AB, AC, and AD, were constructed. In AA stacking, the P atom is positioned directly above the S atom in the SnS_2_ layer and resides at the center of a hexagonal ring in the SnS_2_ layer, while the As atom in the AsP layer is aligned above the S atom in the SnS_2_ layer. In AB stacking, the P atom is located at an above S atom of the SnS_2_ layer and resides at the center of a hexagonal ring in the SnS_2_ layer, and the As atom is located above the Sn atom in the SnS_2_ layer. In the AC configuration, an As atom is placed above an S atom in the SnS_2_ layer, which itself is situated at the center of a hexagonal ring. The P atom is located above the Sn atom of the SnS_2_ layer. The AD stacking features an As atom located below the S atom, and the P atom is aligned above the S atom in the SnS_2_ layer, while the As atom resides at the center of a hexagonal ring in the SnS_2_ layer. The fully relaxed structures are depicted in Fig. 1.

Among them, AA stacking exhibits the lowest interfacial binding energy, indicating it is the most stable configuration. The equilibrium lattice constants, optimized using the GGA-PBE functional, were found to be 3.685 Å for the AsP monolayer, 3.680 Å for the SnS_2_ monolayer, and 3.682 Å for the AA-stacked AsP/SnS_2_ heterostructure, consistent with previously reported values.^14,16–19^ The in-plane lattice parameter of the heterostructure (3.682 Å) results in a minimal lattice mismatch of 0.08% between the constituent layers, confirming the structural feasibility of the AsP/SnS_2_ heterostructure.

To characterize the van der Waals (vdW) interactions in the AsP/SnS_2_ heterostructure, the interfacial binding energy (Δ*E*) was evaluated using the following expression:

4 Δ*E* = (*E*_HS_ − *E*_AsP_ − *E*_SnS_2__)/*S*_0_

Here, Δ*E* denotes the interfacial binding energy, while *E*_HS_, *E*_AsP_, *E*_SnS_2__ and *S*_0_ represent the total energy of the heterostructure, the energy of an isolated AsP monolayer, the energy of an isolated SnS_2_ monolayer, and the interface area, respectively.

**Fig. 2:**
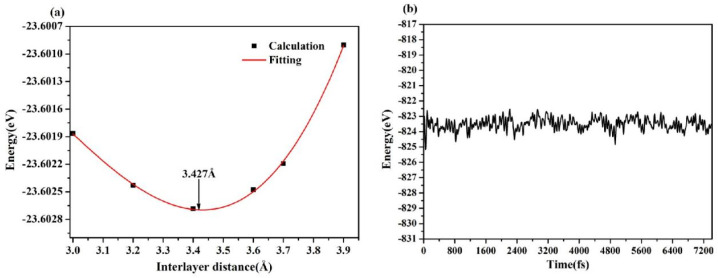
(a) Plot fitting of the interface binding energies of the AsP/SnS_2_ heterostructure as a function of interlayer distance. A fourth-order polynomial has been employed for fitting. (b) Thermal stability of the AsP/SnS_2_ heterostructure.

The calculated binding energy of −9.97 meV Å^−2^ confirms that the interlayer interaction is primarily governed by weak van der Waals forces. The negative value further indicates that the formation of the heterostructure is energetically favorable. As illustrated in Fig. 2(a), the equilibrium interlayer distance between the As atom in the AsP layer and the above S atom in the SnS_2_ layer is 3.427 Å. This separation, which is significantly larger than covalent bonding distances, along with the magnitude of the binding energy, corroborates that the heterostructure is stabilized by vdW interactions, comparable in strength with those found in layered graphite.^43^ In addition, the AIMD simulation of the AsP/SnS_2_ heterostructure for the most favorable stacking AA pattern was also performed to confirm the thermal stability at room temperature, as shown in Fig. 2(b). Obviously, the variation in the total energy of the AsP/SnS_2_ heterostructure was quite small at 7400 fs, indicating that the AsP/SnS_2_ heterostructure is thermally stable at room temperature.

### 3.2 Electronic properties of the AsP/SnS_2_ heterostructure

**Fig. 3:**
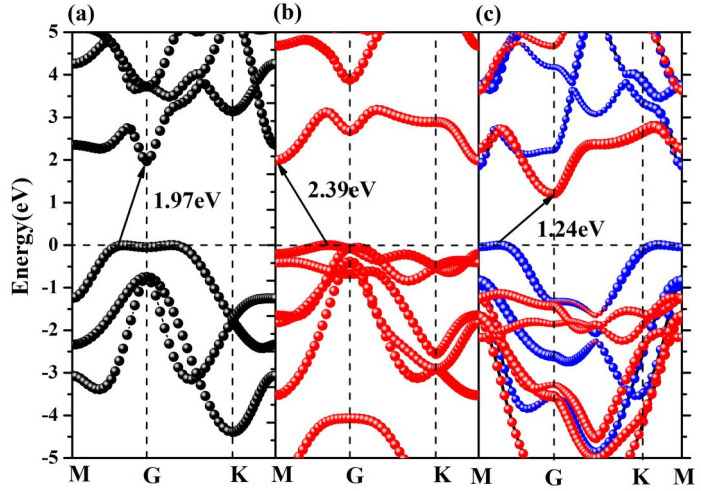
Projection-resolved band structures of the (a) AsP monolayer, (b) SnS_2_ monolayer, and (c) AsP/SnS_2_ heterostructure. The red and blue lines indicate the contributions from the SnS_2_ and AsP layers, respectively.

The projected band structure of the AsP/SnS_2_ heterostructure is presented in Fig. 3. The system is identified as an indirect bandgap semiconductor, with the valence band maximum (VBM) along the M → G path and the conduction band minimum (CBM) located at the G point in the Brillouin zone. The HSE06-calculated band gap of the heterostructure was 1.24 eV, which is smaller than those of the constituent monolayers (AsP (1.97 eV) and SnS_2_ (2.39 eV)) and is in good agreement with previously reported theoretical values of 1.89 eV for AsP^14^ and 2.39 eV for SnS_2_.^19^ Notably, the AsP/SnS_2_ heterostructure exhibits a type-II band alignment, which facilitates the spatial separation of photogenerated electrons and holes across different layers, suppresses carrier recombination, and enhances the light-harvesting efficiency. These properties underscore the potential of vertically stacked heterostructures in band engineering and tailored design of efficient optoelectronic devices.

### 3.3 Bandgap tuning of the AsP/SnS_2_ heterostructure by biaxial strain application

**Fig. 4:**
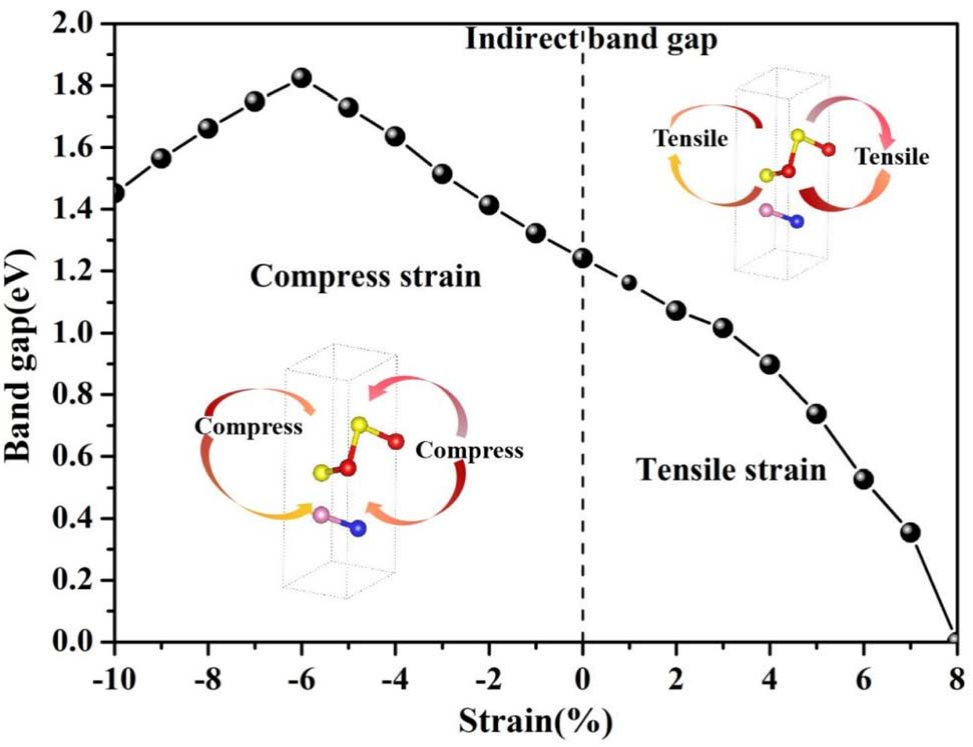
The variation in the band gap of the AsP/SnS_2_ heterostructure at different biaxial strains.

Strain engineering is a fundamental approach for modulating electronic properties by deliberately applying mechanical stress (tensile or compressive) to induce controlled deformation of the lattice. This deformation alters the interatomic bond lengths and angles, leading to modifications in both the crystal field environment and the orbital overlap between neighboring atoms. Such structural changes directly influence the electronic Hamiltonian, notably affecting the energies of the valence and conduction bands. Biaxial strain application is an effective means to modulate the electronic properties of heterostructures. In this work, we systematically examined the influence of biaxial strain ranging from −10% to 8% on the electronic structure of the AsP/SnS_2_ heterostructure, whereby the in-plane lattice parameters of the heterostructure unit cell were controllably modified. All strained configurations were successfully relaxed and found to maintain the hexagonal symmetry, as confirmed by the consistent irreducible Brillouin zone. The evolution of the band gap under applied biaxial strain is presented in Fig. 4. Under compressive strain, reduced interatomic distances typically enhance orbital hybridization, increasing the hopping integrals and generally widening the electronic bandgap while increasing degeneracies due to lowering symmetry. In contrast, tensile strain increases atomic separation, which diminishes orbital overlap and the hopping matrix elements, often resulting in bandgap narrowing. Biaxial strain, when applied uniformly along two in-plane crystallographic directions, results in a symmetric lattice distortion that can be systematically modeled using the density functional theory or tight-binding frameworks to quantify strain-induced electronic structure modifications. Under compressive strain, the non-monotonic variation of the band gap, featuring an initial increase followed by a decrease, can be attributed to the competing effects of orbital hybridization and strain-induced band renormalization. At moderate compressive strains, the enhanced interatomic orbital overlap generally increases crystal field splitting and enlarges the band gap. However, under larger compressive deformation, structural distortions may sufficiently lower the symmetry to activate additional band hybridization pathways, particularly between the valence band maximum (VBM) and the conduction band minimum (CBM), often accompanied by changes in the curvature and effective masses of the bands, leading to a reduction in the gap.

In the case of tensile strain, the monotonic decrease in the band gap primarily stems from the progressive downward shift of the CBM toward the Fermi level. This is caused by reduced interatomic coupling and weakened orbital overlap due to lattice expansion, which diminishes the dispersion of the conduction bands and lowers their energy. When a tensile strain of 8% is applied to the AsP/SnS_2_ heterostructure, the CBM crosses the Fermi level, resulting in a semiconductor-to-metal transition. Notably, this electronic phase transition occurs without the rupture of chemical bonds, indicating preservation of structural integrity while the electronic spectrum is fundamentally altered by strain-induced modifications in the Hamiltonian.

### 3.4 Photocatalyst and absorption behaviors

**Fig. 5:**
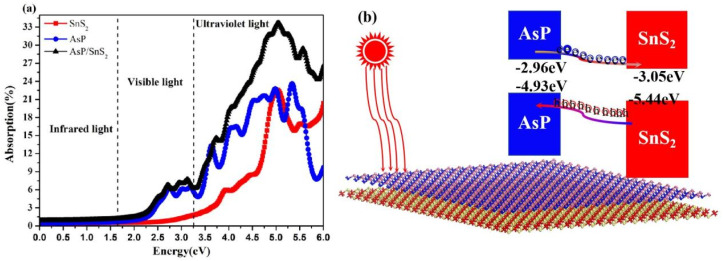
(a) Absorption spectrum of the AsP/SnS_2_ heterostructure and (b) a schematic of the migrating carriers.

The absorption coefficients of the AsP/SnS_2_ heterostructure are calculated and presented in Fig. 5(a). The first prominent absorption peak is located at 2.72 eV, predominantly originating from the AsP layer. Notably, the AsP/SnS_2_ heterostructure exhibits significantly enhanced optical absorption across a broad range compared to the isolated AsP and SnS_2_ monolayers. Furthermore, its absorption spectrum extends well into the visible light region, underscoring its strong potential for application in visible-light optoelectronic devices.

Although the outermost sulfur layers on either side of the AsP/SnS_2_ heterostructure possess identical Pauling electronegativities, the Sn atoms within the SnS_2_ layer exhibit a higher electronegativity (2.44) compared to the As atoms in the adjacent AsP layer (2.14). This electronegativity difference induces a redistribution of electrons across the interface, leading to charge depletion on the AsP side and accumulation on the SnS_2_ side. As a result, a built-in electric field is formed, oriented from the AsP layer toward the SnS_2_ layer. Furthermore, the extent of charge transfer across the interface is correlated with the strength of Sn–As interatomic coupling; a smaller transferred charge corresponds to weaker bonding interactions between the Sn and As atoms.

A schematic illustration of the photogenerated carrier dynamics at the AsP/SnS_2_ interface is depicted in Fig. 5(b). Owing to the type-II band alignment, photogenerated electrons in the conduction band of the AsP layer are transferred to the SnS_2_ layer, driven by a conduction band offset (CBO) of 1.45 eV. Simultaneously, the holes in the valence band of SnS_2_ migrate to the AsP layer, facilitated by a valence band offset (VBO) of 0.57 eV. This efficient spatial separation of electrons and holes, mediated by the built-in electric field, effectively suppresses carrier recombination and enhances the photocatalytic performance of the AsP/SnS_2_ heterostructure.

It is well established that the built-in electric field plays a critical role in determining the lifetime of photogenerated carriers. To elucidate this effect, the built-in electric field induced by ground-state charge transfer was quantitatively evaluated through Bader charge analysis.^44^ The calculation revealed a net charge transfer of 0.02*e* from the AsP layer to the SnS_2_ layer, resulting in a built-in electric field directed from SnS_2_ to AsP. This field facilitates interlayer charge separation and suppresses carrier recombination, thereby significantly enhancing the overall light-harvesting efficiency.

## 4 Conclusions

In summary, we systematically explored the electronic and optical properties of the AsP/SnS_2_ heterostructure using first-principles density functional theory. The hexagonal AsP/SnS_2_ heterostructure is established as a stable semiconductor with an indirect bandgap. The identified type-II band alignment effectively promotes the separation of photogenerated charge carriers, rendering the system particularly suitable for photocatalytic processes. Bader charge analysis indicates a net transfer of 0.02*e* from the AsP layer to SnS_2_, inducing a built-in electric field that further enhances charge separation. In addition, the heterostructure exhibits pronounced optical absorption across the visible spectrum, substantially exceeding those of the individual AsP and SnS_2_ monolayers. These computational insights collectively underscore the potential of the AsP/SnS_2_ heterostructure as a promising candidate for visible-light-driven photocatalysis and advanced optoelectronic applications.

## Conflicts of interest

There are no conflicts to declare.

## Supplementary Material

RA-015-D5RA07850D-s001

RA-015-D5RA07850D-s002

RA-015-D5RA07850D-s003

RA-015-D5RA07850D-s004

RA-015-D5RA07850D-s005

## Data Availability

The data that support the findings of this study are available from the corresponding author (nanx1013@163.com) upon reasonable request. Supplementary information (SI): the densities of AsP and SnS_2_ monolayers and AsP/SnS_2_ heterostructure. See DOI: https://doi.org/10.1039/d5ra07850d.
