# Supplementary figures and images for: Adjustable bandgap of a type-II AsP/SnS2 van der Waals heterostructure using strain: outstanding electronic, optical, and photocatalytic properties
Source: RSC Adv. 2025 Dec 10;15(57):49210–6. doi: 10.1039/d5ra07850d (PMC12690640; doi:10.1039/d5ra07850d)

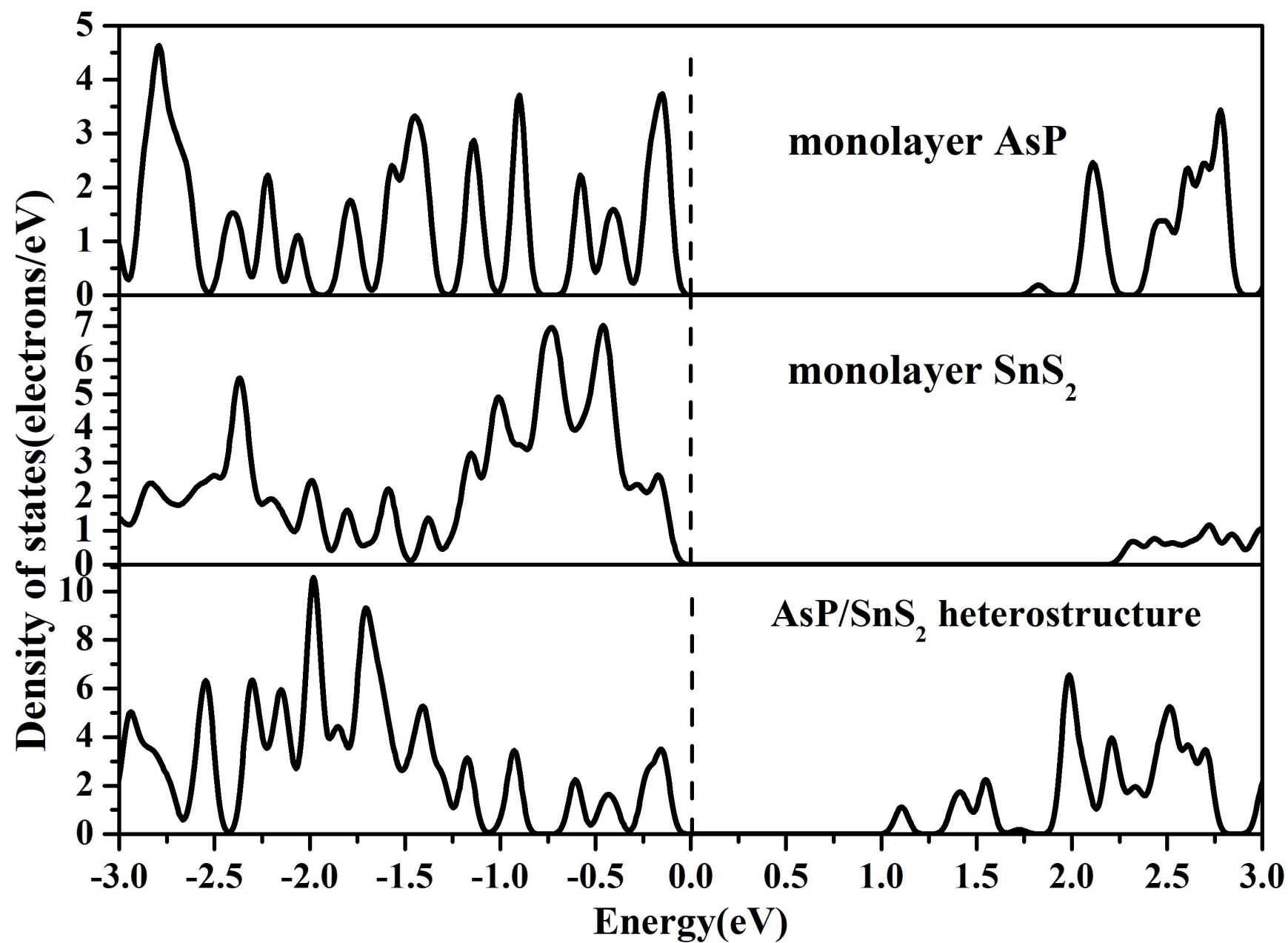

Supplement: RA-015-D5RA07850D-s002 [file RA-015-D5RA07850D-s002.pdf]

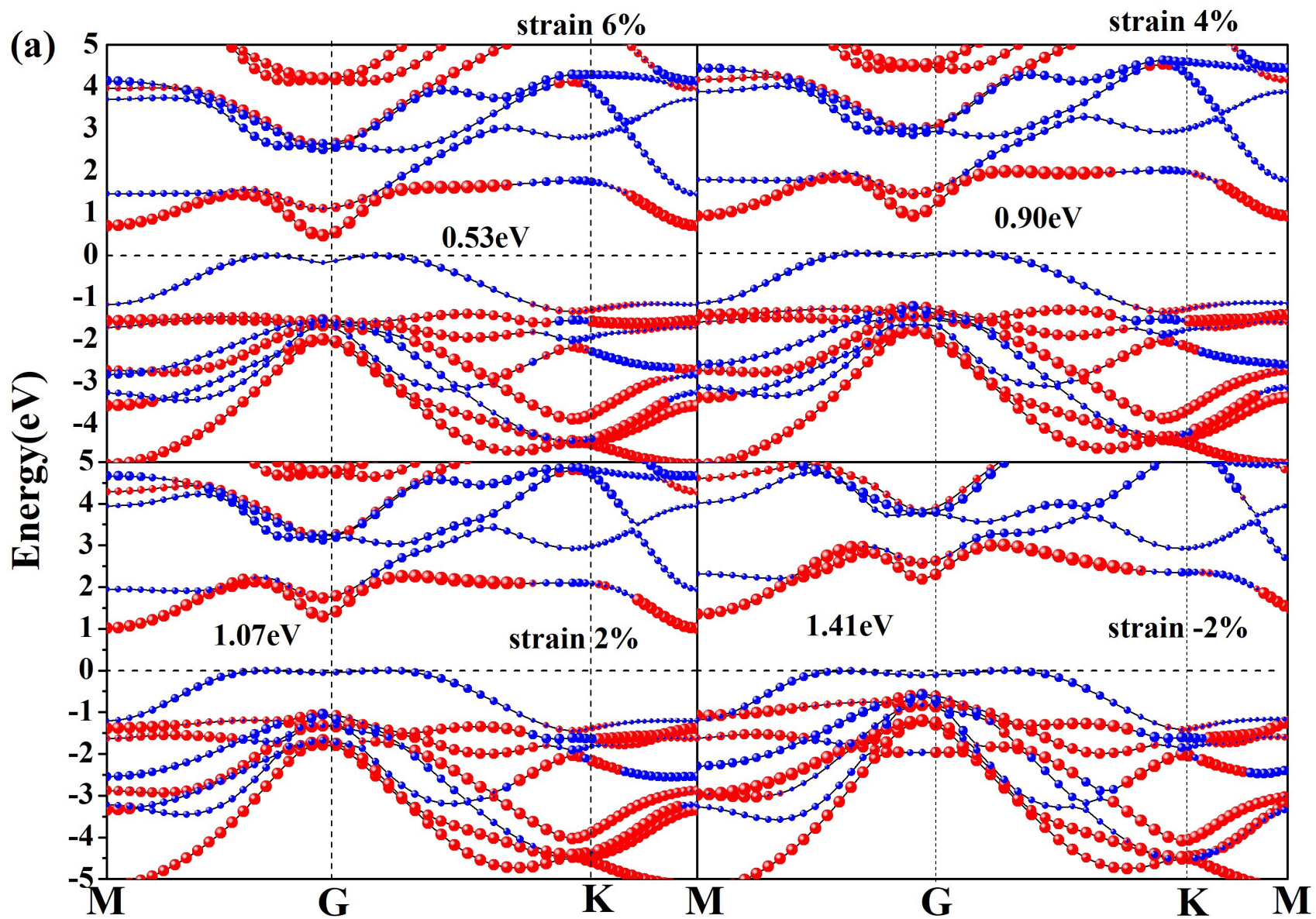

Supplement: RA-015-D5RA07850D-s003 [file RA-015-D5RA07850D-s003.pdf]

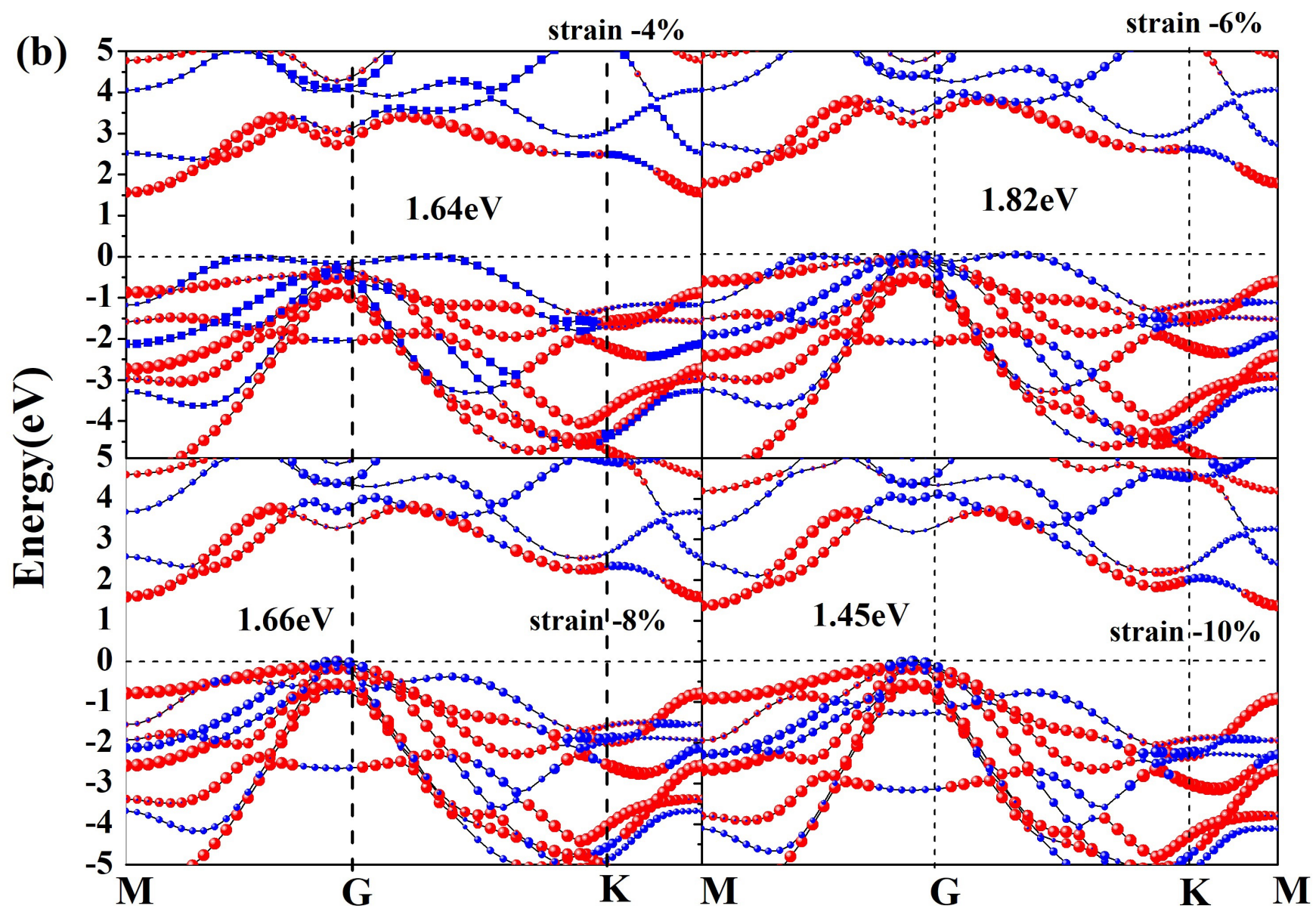

Supplement: RA-015-D5RA07850D-s004 [file RA-015-D5RA07850D-s004.pdf]
