# Supplementary material for: Adjustable bandgap of a type-II AsP/SnS2 van der Waals heterostructure using strain: outstanding electronic, optical, and photocatalytic properties
Source: RSC Adv. 2025 Dec 10;15(57):49210–6. doi: 10.1039/d5ra07850d (PMC12690640; doi:10.1039/d5ra07850d)
Supplement: RA-015-D5RA07850D-s005 [file RA-015-D5RA07850D-s005.pdf]

Supplementary Information (SI) for RSC Advances.
This journal is © The Royal Society of Chemistry 2025

Supplementary Information

Adjustable bandgap of type-II AsP/SnS_2 van der Waals heterostructure by the strain: the outstanding electronic, optical, and photocatalytic properties

Dahua Ren[a], Yao Wen[b], Zhangyang Zhou[a], Ming Du[a], Liushun Wang[a], and Jinqiao Yi[a†]

[a]*College of Intelligent Systems Science and Engineering, Hubei Minzu University, Enshi, China, 44500.*

[b]*School of Physics and Technology, Wuhan University, Wuhan 430072, China*

As evidenced by the projected band structures, the reduction in the band gap arises from a downward shift of the conduction band minimum (CBM) at the G point in the SnS_2 layer and an upward shift of the valence band maximum (VBM) along the M→G path in the AsP layer. In the AsP/SnS_2 heterostructure, as depicted in Fig. 1, the VBM is predominantly contributed by the AsP layer, while the CBM is primarily derived from the SnS_2 layer—where the red and blue curves represent the density of states (DOS) of SnS_2 and AsP, respectively. The spatial separation of the VBM and CBM into the AsP and SnS_2 layers clearly indicates a type-II band alignment in the AsP/SnS_2 heterostructure.

† Corresponding author E-mail address: nanx1013@163.com.

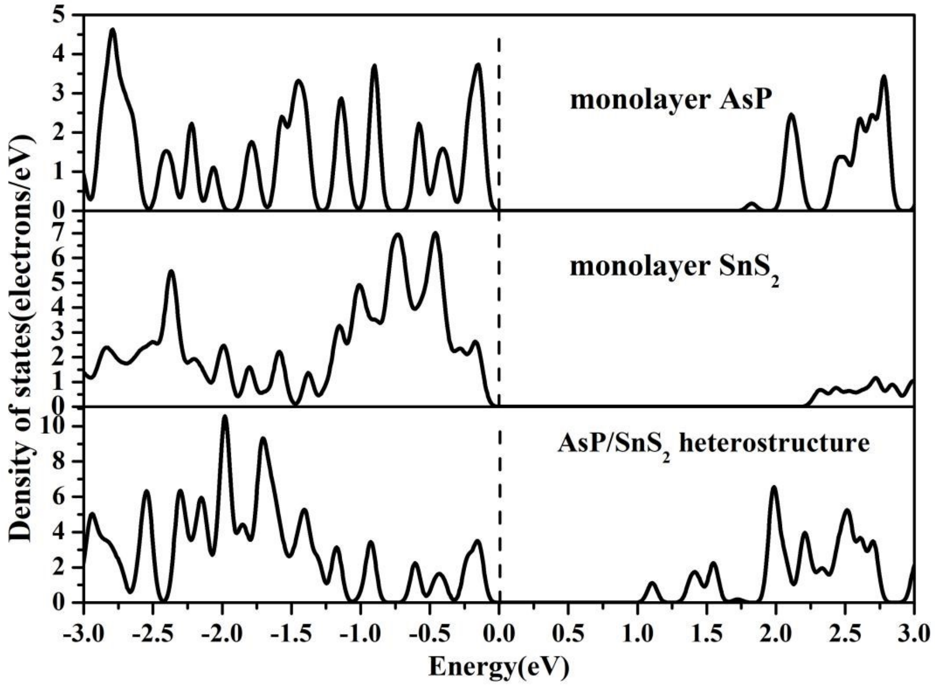

Fig. 1 Projected density of states (PDOS) of AsP monolayer, SnS_2 monolayer, AsP/SnS_2 heterostructure. Fermi level is set to zero as indicated by the black dashed line.

Strain engineering serves as a fundamental approach for modulating electronic properties by deliberately applying mechanical stress—either tensile or compressive—to induce controlled deformation of the lattice. Under compressive strain from 0% to 6%, the band gap initially increases to 1.82 eV. Under compressive strain from 6% to 10%, the band gap decreases from 1.82 eV to 1.45 eV in Fig. 2. In contrast, under tensile strain increasing from 0% to 8%, the band gap monotonically decreases from 1.24 eV to 0 eV in Fig. 2(a). Under compressive strain, the non-monotonic variation of the band gap can be attributed to competing effects of orbital hybridization and strain-induced band renormalization. At moderate compressive strains, the enhanced interatomic orbital overlap generally increases the crystal field splitting and enlarges the band gap. However, under larger compressive deformation, structural distortions may lower symmetry sufficiently to activate additional band hybridization pathways, particularly between the valence band maximum (VBM) and

conduction band minimum (CBM), often accompanied by changes in the curvature and effective masses of the bands, leading to a reduction in the gap. In contrast, tensile strain increases atomic separation, which diminishes orbital overlap and hopping matrix elements, often resulting in bandgap narrowing.

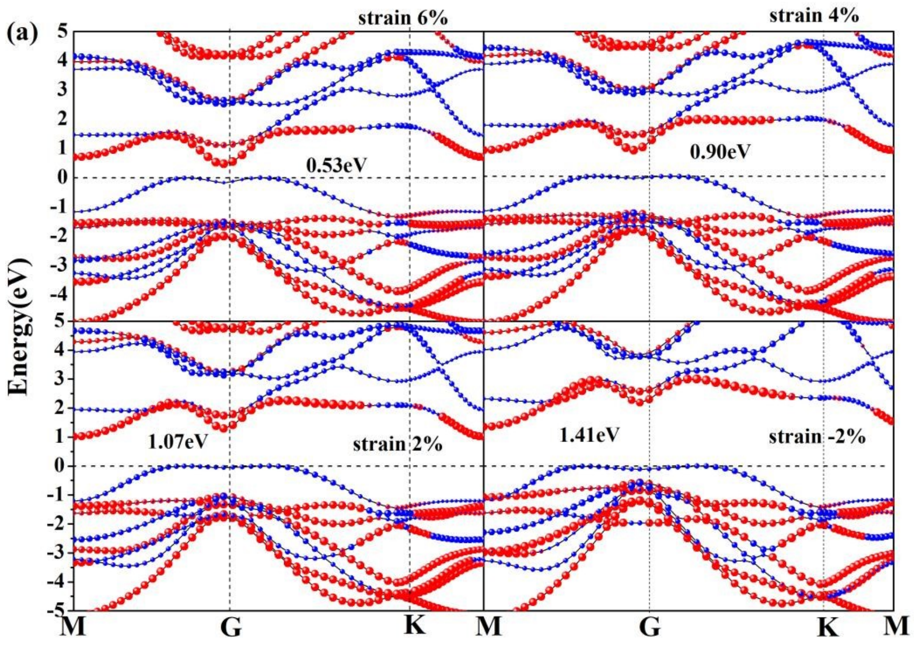

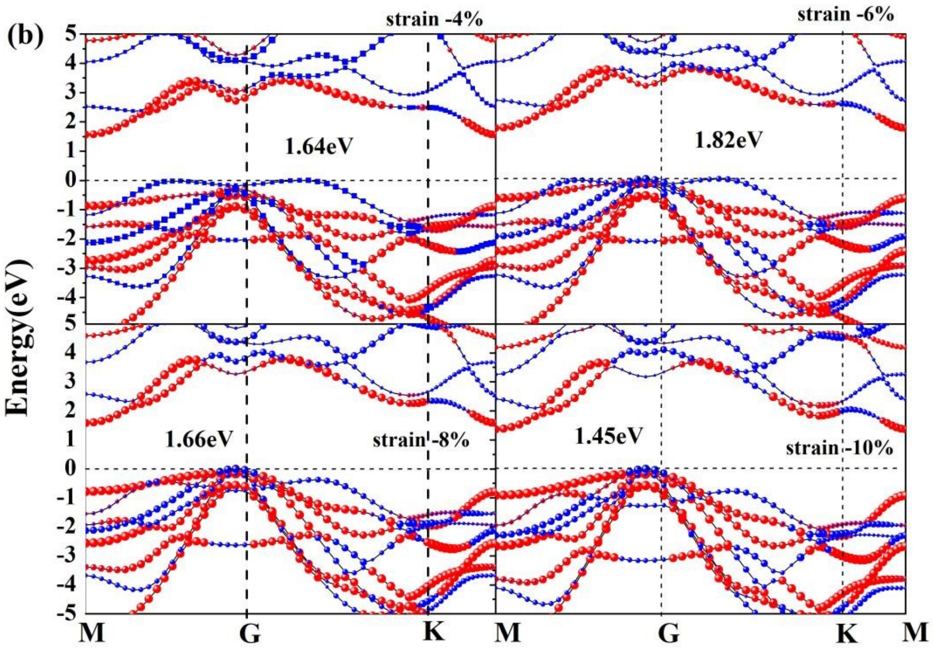

Fig. 2 Band gap of AsP/SnS_2 heterostructure under strain of (a) 6%, 4%, 2% and -2%; (b) -4%, -6%, -8% and -10%.
